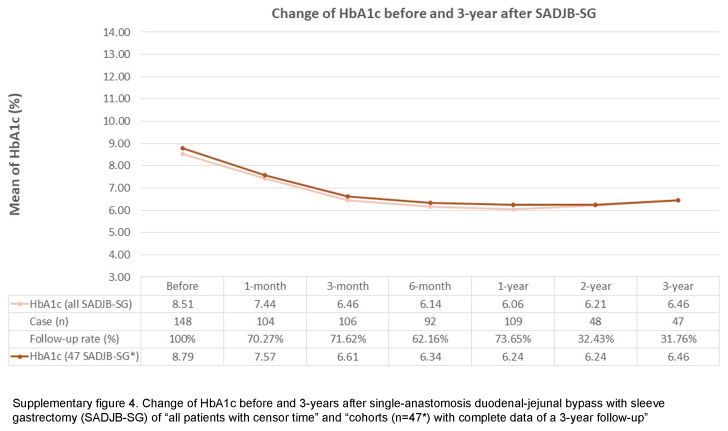# Impact of metabolic bariatric surgery on outcomes and the 10-year risk of major adverse cardiovascular events during a 7-year period: a retrospective cohort study

**DOI:** 10.1097/JS9.0000000000001631

**Published:** 2024-05-21

**Authors:** Hsin-Mei Pan, Wei-Jei Lee, Kong-Han Ser, Tien-Chou Soong, Ming-Hsien Lee, Chien-Hua Lin, Kuo-Feng Hsu

**Affiliations:** a Division of General Surgery, Department of Surgery/Bariatric and Metabolic Surgery and Weight Management Center, Tri‑Service General Hospital, National Defense Medical Center, Taipei; b Medical Weight Loss Center, China Medical University Hsinchu Hospital, Hsinchu; c Bariatric and Metabolic Surgery Center, Ten-Chan General Hospital, Taoyuan; d Center for Weight Loss and Health Management, E-DA Dachang Hospital/College of Medicine, I-Shou University; e Metabolic and Bariatric Surgical Department, Taichung Tzu Chi Hospital, Buddhist Tzu Chi Medical Foundation/Department of Surgery, School of Medicine, Tzu Chi University, Hualien; f Department of Surgery, IRCAD Taiwan, Chang-Bing Show Chwan Memorial Hospital, Taiwan

**Keywords:** comorbidities, diabetes, major adverse cardiovascular events, obesity, one-anastomosis gastric bypass, single-anastomosis duodenal-jejunal bypass with sleeve gastrectomy

## Abstract

**Background:**

Metabolic bariatric surgery offers enduring weight reduction and alleviation of obesity-related comorbidities, including dyslipidemia, diabetes, hypertension, and major adverse cardiovascular events (MACE). Long-term data on one-anastomosis gastric bypass (OAGB) and single-anastomosis duodenal-jejunal bypass with sleeve gastrectomy (SADJB-SG) is lacking, necessitating this investigation.

**Materials and Methods:**

In this multicenter prospectively-collected retrospective observational study, 830 adult Taiwanese patients (682 OAGB, 148 SADJB-SG) who underwent surgery from 1 January 2011 to 31 December 2017, were initially identified. Following protocol, 224 patients (177 OAGB, 47 SADJB-SG) with complete follow-up data at various intervals up to 3 years after surgery were included in the final analysis. The study’s primary focus is to evaluate the long-term safety, efficacy, and durability of OAGB and SADJB-SG in promoting weight loss and diabetes remission. Additionally, changes in 10-year and lifetime risks of MACE before and 3-year after surgery are assessed using Taiwan MACE risk prediction model and the China-PAR project model.

**Results:**

SADJB-SG patients exhibit higher diabetes prevalence, lower BMI, and more severe diabetes compared to OAGB. Both groups demonstrate significant improvements in BMI, diabetes, hypertension, and dyslipidemia three years after surgery, with the most substantial improvements occurring in the second year. The Taiwan MACE risk model reveals a significant reduction in 10-year MACE and stroke risks for both groups. The China-PAR project model indicates a synchronized reduction in atherosclerotic cardiovascular disease 10-year and lifetime risk in both OAGB and SADJB-SG groups.

**Conclusions:**

OAGB and SADJB-SG exhibit sustained improvements in weight reduction and obesity-related comorbidities over 3 years after surgery. Notably, both procedures contribute to a substantial reduction in 10-year MACE, stroke, and atherosclerotic cardiovascular disease risks. These findings underscore the efficacy of OAGB and SADJB-SG in the context of metabolic bariatric surgery.

## Graphical abstract

**Figure:**
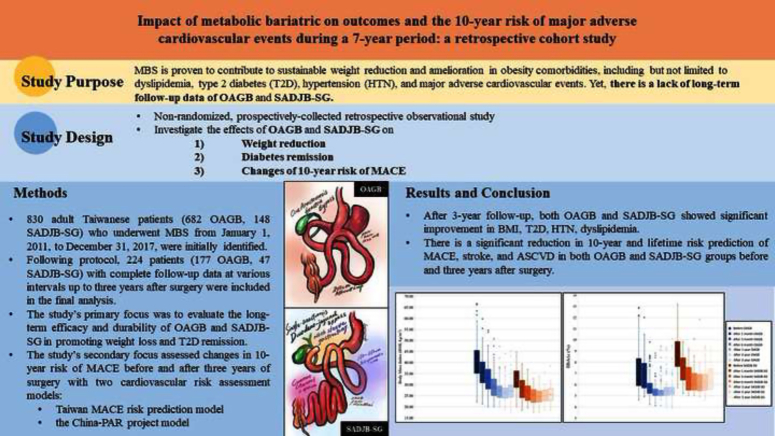

## Introduction

Highlights

- Our study investigates two novel metabolic bariatric surgery procedures, one-anastomosis gastric bypass (OAGB) and single-anastomosis duodenal-jejunal bypass with sleeve gastrectomy (SADJB-SG), aiming to understand their long-term treatment effects on obesity, diabetes, obesity-related comorbidities, 10-year and lifetime risks of major adverse cardiovascular events in Asian population.
- Both OAGB and SADJB-SG provide sustained weight reduction, remission of diabetes, and improvements in hypertension and dyslipidemia, persisting for at least 3 years after surgery.
- OAGB and SADJB-SG effectively mitigate cardiovascular risks in Taiwanese patients over the long-term. This conclusion is supported by two risk assessment tools developed using Taiwan’s national healthcare data and a dataset from China, respectively.

Obesity has emerged as a longstanding global health crisis, substantially amplifying the overall disease burden^1^. Therefore, it is imperative to implement efficacious and enduring interventions for individuals grappling with obesity, with the overarching objective of mitigating the likelihood of developing cardiovascular ailments and subsequent mortality^2^. Among the array of multimodal approaches to address obesity, metabolic bariatric surgery (MBS) has demonstrated its efficacy in achieving sustainable weight loss and alleviating associated comorbidities, encompassing, but not limited to, dyslipidemia, type 2 diabetes (T2D), hypertension (HTN), obstructive sleep apnea, osteoarthritis (OA), fatty liver disease, nonalcoholic steatohepatitis (NASH), as well as various cardiovascular conditions such as coronary artery disease, atrial fibrillation, and heart failure^3–11^.

According to the 8th Global Registry Report of the International Federation for the Surgery of Obesity and Metabolic Disorders (IFSO) in 2023^12^, the primary MBS procedures globally comprise sleeve gastrectomy (SG) (63.3%), Roux-en-Y gastric bypass (RYGB) (28.8%), one-anastomosis gastric bypass (OAGB) (4.1%), and other procedures (5.8%). Regarding revisional MBS procedures, the distribution includes SG (23.8%), RYGB (42.8%), and OAGB (9.0%). Yet, the mostly studied MBS procedures in the existed literature are SG, RYGB, adjustable gastric banding (AGB), vertical banded gastroplasty, and duodenal switch^13^. Thus, further research is warranted to ascertain the efficacy and durability of OAGB and other less commonly performed procedures, such as single-anastomosis duodenojejunal bypass with sleeve gastrectomy (SADJB-SG). Therefore, the paucity of evidence regarding OAGB and SADJB-SG necessitates further research.

The primary objective of our investigation is to assess the long-term impact and sustainability of weight loss as well as the remission of T2D up to 3 years following MBS in relation to OAGB and SADJB-SG. The secondary aim is to appraise alterations in the 10-year and lifetime risks of major adverse cardiovascular events (MACE), a pivotal concern in the timely management of obesity and its associated comorbidities, before and after 3 years of OAGB and SADJB-SG in the patient population in Taiwan.

## Materials and methods

### Study design

This study is a multicenter prospectively-collected retrospective observational study investigating the effects of OAGB and SADJB-SG on weight reduction, diabetes remission, and changes of 10-year and lifetime risks of MACE in patients with obesity in Taiwan.

**Figure 1:**
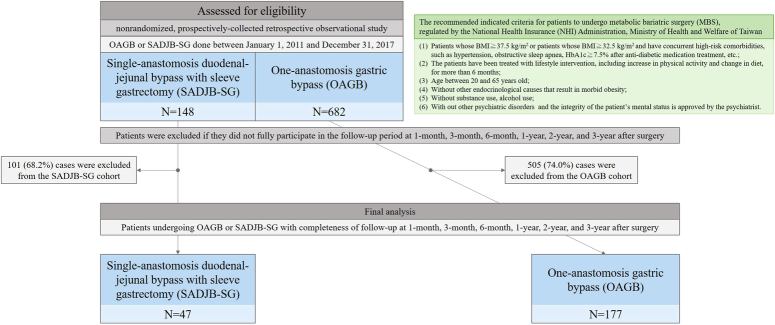
The inclusion and exclusion process of the final analyzed groups: one-anastomosis gastric bypass (OAGB) and single-anastomosis duodenal-jejunal bypass with sleeve gastrectomy (SADJB-SG).

This study was carried out in the bariatric and metabolic surgery centers of tertiary hospitals and was approved by the human research review board (MSIRB2014009, B202105089). The work has been reported in line with the strengthening the reporting of cohort, cross-sectional, and case–control studies in surgery (STROCSS) criteria^14^ (Supplemental Digital Content 1, http://links.lww.com/JS9/C630). As shown in Figure 1, a total of 830 adult Taiwanese patients (age between 20 and 65 years) undergoing OAGB (*n*=682) and SADJB-SG (*n*=148) between 1 January 2011 and 31 December 2017 were identified. Patients were excluded if they did not fully participate in the follow-up period at 1-month, 3-month, 6-month, 1-year, 2-year, and 3-year after surgery with the primary concern of the completeness of data. Following the protocol, a total of 224 patients undergoing OAGB (*n*=177, 26.0% of the original OAGB cases) and SADJB-SG (*n*=47, 31.8% of the original SADJB-SG cases) were included into the final analysis.

### Patient selection and shared-decision-making of choice of procedures

The inclusion criteria of MBS is regulated by the National Health Insurance (NHI) Administration, Ministry of Health and Welfare of Taiwan. The criteria includes: (1) patients whose BMI≧37.5 kg/m^2^ or patients whose BMI≧32.5 kg/m^2^ and have concurrent high-risk comorbidities, such as HTN, obstructive sleep apnea, HbA1c ≧7.5% after antidiabetic medication treatment, etc.; (2) the patients have been treated with lifestyle intervention, including increase in physical activity and change in diet, for more than 6 months; (3) age between 20 and 65 years old; (4) without other endocrinological causes that result in morbid obesity; (5) without substance use and alcohol use; (6) without psychiatric disorders and the integrity of the patient’s mental status is approved by the psychiatrist.

Once the patient is eligible for MBS, multidisciplinary shared-decision-making discussion is carried out regarding choice of MBS, OAGB, or SADJB-SG. On the one hand, OAGB is recommended for patients with greater BMI due to following concerns: (1) SADJB-SG is less suitable for patients with greater BMI given the potentially-increased risk resulting from the complexity of duodenal division; (2) OAGB can accomplish effective weight reduction and remission of metabolic disorders for patients with obesity despite the simplicity of the procedure. On the other hand, SADJB-SG is primarily reserved for patients with less severe BMI and/or concurrent, more serious T2D in order to counterbalance the safety and the efficacy of metabolic and bariatric outcomes. All informed consents are obtained from the participants.

### Study measures

Various variables and relevant medical history are collected and measured to determine the demographic characteristics of both surgical groups, to demonstrate perioperative safety of both procedures, and to quantify the change of essential laboratory findings, including blood sugar, blood pressure, and lipid profile, before and after the patients underwent either OAGB or SADJB-SG. In addition, the definition of following diseases, dyslipidemia, diabetes mellitus, and HTN, is defined as described: (1) dyslipidemia encompasses elevated levels of total cholesterol (T-CHO), low-density lipoprotein cholesterol (LDL-C), triglycerides (TG), and reduced levels of high-density lipoproteins cholesterol (HDL-C); (2) patients whose HbA1c levels are greater than 6.5% are defined as diabetes and those HbA1c levels are between 5.7 and 6.4% are defined as prediabetes, according to American Diabetes Association (ADA).

The items relevant to demographic characteristics includes: age (year), sex (male or female), height (centimeter, cm), weight (kilogram, kg), waist circumference (centimeter, cm), (BMI, weight in kilograms divided by height in meters squared, kg/m^2^). The perioperative variables include: operative time (minute), intraoperative blood loss (milliliter), timing of postoperative flatus passage (day), length of hospital stay (day), complication (overall, major, and minor). The essential laboratory findings include: SBP (mmHg), DBP (mmHg), T-CHO (mg/dl), HDL (mg/dl), LDL (mg/dl), TG (mg/dl), alanine aminotransferase (ALT, mg/dl), gamma glutamyl transferase (GGT, mg/dl), albumin (g/dl), glycated hemoglobin (HbA1c, %), fasting blood sugar (mg/dl), insulin (μU/ml), C-peptide (mg/ml), hemoglobin (Hb, g/dl), mean corpuscular volume (MCV, fl), serum iron (μg/dl), total iron binding capacity (TIBC, μg/dl), ferritin (mg/ml).

## Procedures^15^

### One-anastomosis gastric bypass (OAGB)

**Figure 2:**
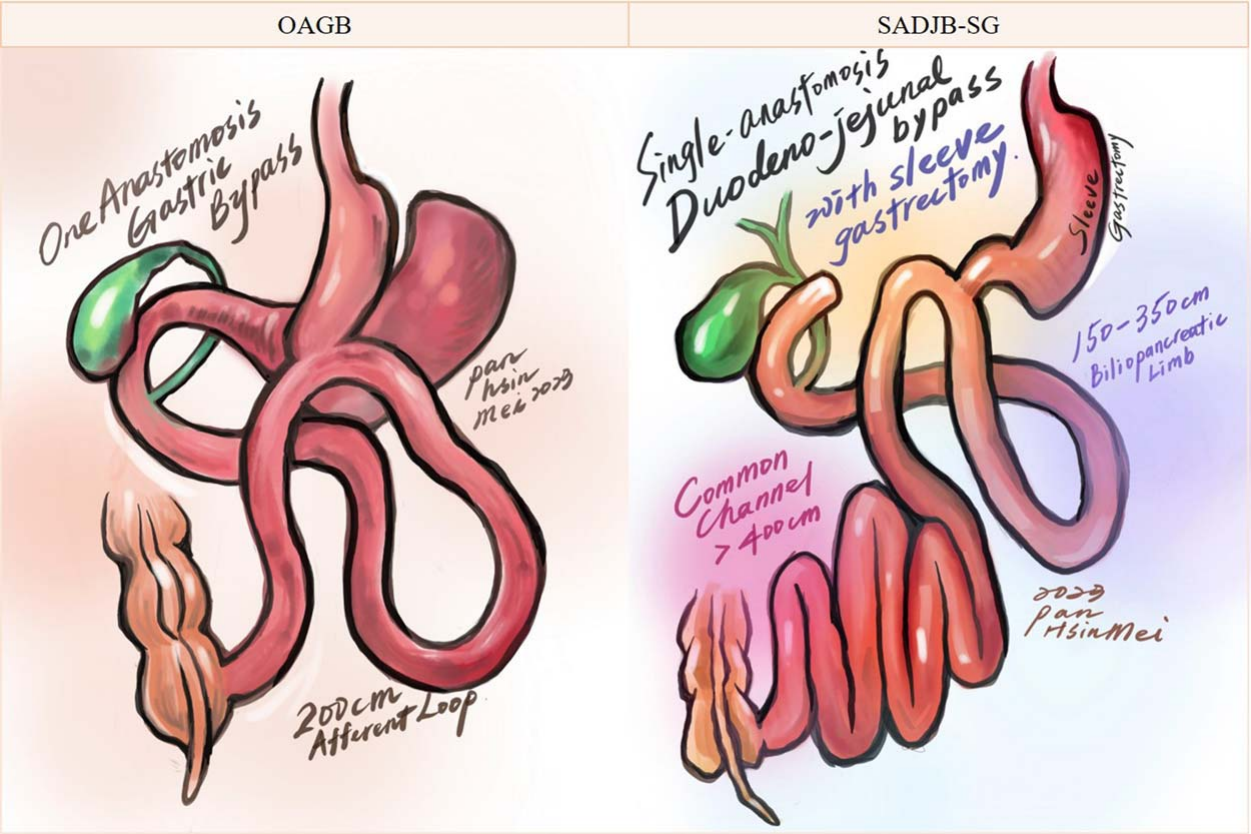
Illustration of surgery: one-anastomosis gastric bypass (OAGB) and single-anastomosis duodenal-jejunal bypass with sleeve gastrectomy (SADJB-SG).

The execution of OAGB (Fig. 2, left) involves an initial step with a 2 cm-wide long sleeve gastric tube along the lesser curvature, extending from the antrum to the angle of His through the continuous application of staplers. Subsequently, seromuscular sutures are employed to reinforce the staple lines. Following this, a Billroth II type loop gastroenterostomy is established with the intestine positioned 150–250 cm distal to the ligament of Treitz. The afferent loop is then anchored to the proximal portion of the gastric tube to mitigate bile reflux. Lastly, the gastroenterostomy is secured to impede loop rotation^16^.

### Single-anastomosis duodenojejunal bypass with sleeve gastrectomy (SADJB-SG)

SADJB-SG (Fig. 2, right)^17^ is executed in the French position utilizing a standard 5-port laparoscopic technique. The SG is completed with a 36 Fr. bougie and a linear stapler. Subsequently, dissection is extended through the lower segment and the posterior wall of the duodenum above the gastroduodenal artery. The duodenum is then divided, preserving the right gastric artery and supra-duodenal vessels. The length of the alimentary limb is tailored based on the patient’s BMI, employing a 150 cm biliopancreatic limb for patients with BMI <35 kg/m² and a 200–350 cm alimentary limb for those with BMI >35 kg/m². The selected loop is ascended antecolically without omental division, and a stapler is utilized to create an isoperistaltic side-to-side duodenojejunal anastomosis. Finally, the stapler defect is closed with a 2-layer running absorbable suture, and an air leak test is routinely conducted.

### Taiwan MACE risk prediction model

The first model, published in 2022, is a 10-year MACE risk prediction model developed by Chang *et al*.^18^ in Taiwan. The MACE, including coronary heart disease (CHD) and stroke, used in this prediction model were extracted from the NHI database and death registry; meanwhile, only hospitalization or death due to CHD or stroke was considered. Furthermore, given the structured registry based on international classification of disease of NHI database, CHD is defined as ICD-9: 410-414 or ICD-10: I20-I25; stroke is defined as ICD-9:430-438 or ICD-10: I60-I69. The variables used in the prediction model include: sex (female or male), age (year), height (cm), weight (kg), waist circumference (cm), SBP (mmHg), T-CHO (mg/dl), HDL-C (mg/dl), LDL-C (mg/dl), TG (mg/dl), fasting blood sugar (mg/dl), diagnosed with diabetes/ HTN or not, and having habit of smoking or not.

### The China-PAR project model for predicting 10-year and lifetime ASCVD risk

The second model is the China-PAR project model (Prediction for Atherosclerotic Cardiovascular Disease Risk in China)^19^. This model is specifically formulated to anticipate the 10-year and lifetime probabilities of atherosclerotic cardiovascular disease (ASCVD) within the context of the Chinese population. Due to historical and geographical affinities, the Taiwanese population is deemed analogous to the Chinese population. Consequently, we have opted to employ the China-PAR project model for assessing both the lifetime and 10-year risks of ASCVD in our patient cohorts. In this risk assessment tool, ASCVD is defined as nonfatal acute myocardial infarction (MI), CHD death, or fatal or nonfatal stroke. The China-PAR project model, recognizing the limitations of existing models designed for predominantly white populations, addresses the need for precise ASCVD risk assessment in the Chinese population. Compared to American guidelines, the China-PAR equations exhibited superior performance, offering effective tools for accurate 10-year ASCVD risk prediction in the Chinese population, thereby enhancing primary prevention and cardiovascular disease management.

### Application of risk predicting models in our study

In our study, the parameters, analyzed with these two risk assessment tools, are extracted from two point-in-time, one is preoperative data, while the other is the data 3 years after MBS. Following, we enter the variables into the public calculators of both tools (Taiwan MACE risk prediction model and China-PAR project model). Then, the risk of each patient of both surgical groups before and after 3-year of MBS is computed. The risk stratification, in line with the WHO is defined as below: <10% is defined as low-risk, 10% to <20% is defined as moderate-risk, ≧20% is defined as high-risk. We document the predicted risk of each patient on two point-in-time and further evaluate the change in the risk of 10-year and lifetime MACE.

### Statistics

Data are summarized with descriptive statistics to present the main features of our dataset, including measures of central tendency (mean, median) and dispersion (SD, interquartile range). A meticulous data cleaning process is implemented to identify and address missing values, outliers, and inconsistencies. The primary outcome analysis focuses on the resolution of T2D and BMI before and 3-year after OAGB or SADJB-SG. The secondary outcome, CVD risk, is estimated using the Taiwan MACE risk prediction model and China-PAR project model. Our research is based on the cohort with completion of 3-year follow-up while the statistics were analyzed with Microsoft Excel and IBM SPSS version 28^20^. Statistical significance is defined as *P*-value <0.05.

## Results

### Baseline characteristics

In this study, we initially included 682 cases of OAGB and 148 cases of SADJB-SG (Table 1). Discernible distinctions were observed across demographic, anthropometric, and perioperative parameters. The SADJB-SG cohort exhibits a slightly higher yet similar mean age compared to the OAGB group (42.03 vs. 39.91 years). Differences in obesity parameters are also identified, including body weight (93.47 vs. 111.10 kg, *P*<0.05), waist circumference (108.29 vs. 119.25 cm, *P*<0.05), and BMI (34.19 vs. 40.32 kg/m², *P*<0.05). These findings suggest that the OAGB cohort exhibits severer obesity. Regarding perioperative factors, the SADJB-SG cohort exhibits significantly prolonged operative time, heightened blood loss, delayed flatus passage, and an extended hospital stay (*P*<0.05), highlighting the inherent challenges associated with the complexity of SADJB-SG. Disparity in overall complication (Clavien–Dindo classification system) of both groups is also observed, with the SADJB-SG cohort exhibiting a higher incidence compared to OAGB (14.9 vs. 1.9%, *P*<0.05).

**Table 1:** Overall demographic and clinical characteristics of the two surgery groups: OAGB and SADJB-SG.

	OAGB	SADJB-SG	*P*
Total case number (*n*)	682	148	
Age (year)	39.91±11.43	42.03±10.87	< 0.05
Sex			
Male, *n* (%)	286 (41.9%)	52 (35.1%)	0.1209
Female, *n* (%)	396 (58.1%)	96 (64.9%)	
Obesity parameters			
Body weight (kg)	111.10±26.80	93.47±19.87	< 0.05
Waist circumference (cm)	119.25±17.49	108.29±13.60	< 0.05
BMI (kg/m^2^)	40.32±7.92	34.19±5.93	< 0.05
Perioperative parameters			
Operative time (min)	157.01±38.30	189.63±32.12	< 0.05
Blood loss (ml)	38.53±28.43	43.53±17.91	< 0.05
Flatus passage (day)	1.69±0.56	1.84±0.47	< 0.05
Hospital stay (day)	3.15±2.99	4.97±5.11	< 0.05
Overall complication, *n* (%)	13 (1.9%)	22 (14.9%)	< 0.05
Minor complication			
Grade 1	4 (0.6%)	6 (4.1%)	
Grade 2	5 (0.7%)	7 (4.7%)	
Grade 3a	1 (0.1%)	2 (1.4%)	
Major complication			
Grade 3b	2 (0.3%)	6 (4.1%)	
Grade 4	0 (0.0%)	1 (0.7%)	
Grade 5	1 (0.1%)	0 (0.0%)	

OAGB, one anastomosis gastric bypass; SADJB-SG, single-anastomosis duodenojejunal bypass with sleeve gastrectomy.

The major complications of OAGB include: postoperative leakage with intra-abdominal infection and afferent loop syndrome, postoperative hematochezia, postoperative acute renal injury. The major complications of SADJB-SG include: postoperative leakage of anastomosis site, intraoperative severe blood loss, postoperative acute respiratory distress syndrome, postoperative wound infection with methicillin-resistant Staphylococcus aureus, and postoperative acute internal bleeding.

These findings contribute valuable insights into the comparative clinical outcomes and demographic profiles associated with OAGB and SADJB-SG, elucidating potential factors that may inform surgical decision-making in the context of bariatric interventions. However, considering the thoroughness of the data collected over the 3-year follow-up period, we include only 177 cases of OAGB and 47 cases of SADJB-SG in the analyzed cohort (Table 2). Table 2 presents a comparative analysis between patients undergoing OAGB and SADJB-SG procedures, with a focus on various health parameters. Among the key findings, patients who underwent SADJB-SG have a significantly higher prevalence of T2D compared to the OAGB group (87.2 vs. 55.4%, *P* < 0.05). Additionally, significant differences are observed in BMI, HbA1c levels, HOMA-IR, fasting blood sugar, insulin levels, C-peptide, and SBP levels, all favoring the SADJB-SG cohort with higher values (*P*<0.05). Apart from the above findings, the two groups show comparable values in terms of diastolic blood pressure, lipid profile, liver function, and various hematological parameters. These findings highlight distinctions in metabolic and cardiovascular parameters between OAGB and SADJB-SG cohorts, offering valuable insights for bariatric surgeons when selecting procedures. The differences in diabetic and metabolic markers underscore the need to customize surgical interventions based on individual patient profiles.

**Table 2:** Preoperative laboratory data of the final analyzed groups: OAGB and SADJB-SG.

	OAGB	SADJB-SG	*P*
Case number (*n*)	177	47	
T2D, *n* (%)	98 (55.4%)	41 (87.2%)	< 0.05
BMI (kg/m^2^)	41.37±8.20	32.55±5.66	< 0.05
Diabetic parameters			
HbA1c (%)	7.30±1.85	8.79±1.76	< 0.05
HOMA-IR	7.71±11.18	11.23±14.94	< 0.05
Fasting blood sugar (mg/dl)	131.05±56.94	186.26±69.35	< 0.05
Insulin (μU/ml)	23.20±23.38	25.61±39.13	< 0.05
C-peptide (mg/ml)	3.97±2.79	3.14±1.86	< 0.05
Blood pressure			
SBP (mmHg)	143.62±17.97	136.22±15.44	< 0.05
DBP (mmHg)	89.48±14.59	88.78±12.05	0.7404
Lipid profile			
T-CHO (mg/dl)	209.31±158.90	198.05±34.31	0.3911
HDL (mg/dl)	43.00±9.82	41.84±9.14	0.4605
LDL (mg/dl)	123.75±37.52	114.80±30.35	0.1014
TG (mg/dl)	227.30±351.66	270.67±200.53	0.2799
Liver function			
ALT (IU/l)	58.99±53.98	46.85±42.83	0.1098
GGT (IU/l)	60.12±74.37	54.45±46.97	0.5352
Albumin (g/dl)	4.33±0.30	4.39±0.30	0.2653
Hb (g/dl)	14.55±1.66	14.34±1.60	0.1569
MCV (fl)	83.82±8.07	81.58±9.88	0.1569
Serum iron (μg/dl)	84.17±32.19	87.07±33.02	0.6211
TIBC (μg/dl)	353.07±47.40	332.73±66.22	0.6211
Ferritin (ng/ml)	167.23±154.56	193.81±161.67	0.0724

ALT: alanine aminotransferase; DBP: diastolic blood pressure; GGT: gamma glutamyl transferase; HbA1c: glycated hemoglobin; HDL: high-density lipoprotein; HOMA-IR: homeostatic model assessment for insulin resistance; LDL: low-density lipoprotein; MCV: mean corpuscular volume; OAGB: one anastomosis gastric bypass; SADJB-SG: single-anastomosis duodenojejunal bypass with sleeve gastrectomy; SBP: systolic blood pressure; T-CHO: total cholesterol; TG: triglyceride; TIBC: total iron binding capacity.

### Change in BMI, diabetes, HTN, and dyslipidemia before and after surgery

The primary goal in this study is to evaluate the enduring effects and sustainability of weight loss, as well as the remission of T2D, for up to three years after SADJB-SG and OAGB. Tables 3 and 4 illustrate the alterations in BMI, diabetes, HTN, and dyslipidemia before and after surgery for both cohorts.

**Table 3:** Comparison of laboratory data before, 1-month, 3-month, 6-month, 1-year, 2-year, and 3-year after OAGB (*n*=177).

	Before	1-month	3-month	6-month	1-year	2-year	3-year	*P* *
BMI (kg/m^2^)	41.37±8.20	35.98±7.36	33.11±6.55	29.49±5.60	27.34±4.51	26.45±3.96	27.25±4.26	< 0.05
Diabetic parameters								
HbA1c (%)	7.30±1.85	6.86±1.43	5.81±1.00	5.43±0.69	5.51±0.98	5.44±0.79	5.50±0.82	< 0.05
HOMA-IR	7.71±1.85	1.73±2.04	1.56±1.57	1.03±0.94	1.18±3.09	2.35±14.69	1.14±0.67	< 0.05
Fasting blood sugar (mg/dl)	131.05±56.94	122.68±44.67	102.60±27.09	96.15±22.71	97.02±25.45	95.75±19.56	94.64±18.31	< 0.05
Insulin (μU/ml)	23.20±23.38	10.26±7.46	7.85±5.43	6.01±3.31	6.34±10.67	5.38±3.69	5.38±2.71	< 0.05
C-peptide (mg/ml)	3.97±2.79	2.74±1.63	2.16±0.96	1.79±0.66	1.53±1.27	1.46±0.73	1.53±0.87	< 0.05
Blood pressure								
SBP (mmHg)	143.62±17.97	139.88±18.32	136.42±20.95	133.19±18.36	125.52±17.55	127.97±18.37	128.35±19.42	< 0.05
DBP (mmHg)	89.48±14.59	85.45±12.84	83.26±15.16	80.53±13.39	75.42±12.49	75.74±12.12	74.00±12.40	< 0.05
Lipid profile								
T-CHO (mg/dl)	209.31±158.90	194.39±38.78	185.77±40.34	179.00±34.05	165.01±34.65	164.58±31.51	162.31±29.86	< 0.05
HDL (mg/dl)	43.00±9.82	33.79±6.91	36.00±7.53	42.09±9.02	49.37±10.56	52.68±13.02	53.10±11.81	< 0.05
LDL (mg/dl)	123.75±37.52	134.78±34.96	124.92±32.18	118.30±31.40	100.01±30.60	93.36±29.99	96.21±25.38	< 0.05
TG (mg/dl)	227.30±351.66	162.70±80.10	127.25±62.77	99.38±43.66	85.89±44.72	86.73±79.88	80.63±48.13	< 0.05
Liver function								
ALT (IU/l)	58.99±53.98	53.95±46.93	37.57±26.51	32.24±50.82	33.26±22.83	35.49±37.02	30.87±28.93	< 0.05
GGT (IU/l)	60.12±74.37	33.80±21.25	31.94±28.81	34.68±71.27	29.25±51.55	39.94±104.23	38.63±116.17	0.2325
Albumin (g/dl)	4.33±0.30	5.15±6.46	4.13±0.28	4.15±0.30	4.14±0.31	4.07±0.35	4.13±0.33	0.0653
Hb (g/dl)	14.55±1.66	13.85±1.41	14.18±7.39	13.08±1.99	12.80±2.25	12.72±2.05	12.89±6.22	< 0.05
MCV (fl)	83.82±8.07	83.17±6.78	84.03±6.23	85.05±6.46	84.41±8.26	83.79±8.24	82.66±9.99	0.3470
Serum iron (μg/dl)	84.17±32.19	68.46±37.23	63.90±24.11	70.02±32.70	76.12±45.30	77.75±51.98	68.98±44.83	< 0.05
TIBC (μg/dl)	353.07±47.40	283.83±38.52	286.89±49.83	300.50±54.46	349.59±338.81	321.09±72.36	337.76±69.81	0.1399
Ferritin (ng/ml)	167.23±154.56	252.41±205.35	189.11±166.67	184.05±202.26	148.99±170.91	111.78±177.60	95.05±144.69	< 0.05

* Denotes comparison between values ​​before surgery and three years after surgery.

ALT, alanine aminotransferase; DBP, diastolic blood pressure; GGT, gamma glutamyl transferase; HbA1c, glycated hemoglobin; HDL, high-density lipoprotein; HOMA-IR, homeostatic model assessment for insulin resistance; LDL, low-density lipoprotein; MCV, mean corpuscular volume; OAGB, one anastomosis gastric bypass; SBP, systolic blood pressure; T-CHO, total cholesterol; TG, triglyceride; TIBC, total iron binding capacity.

**Table 4:** Comparison of laboratory data before, 1-month, 3-month, 6-month, 1-year, 2-year, and 3-year after SADJB-SG (*n*=47).

	Before	1-month	3-month	6-month	1-year	2-year	3-year	*P* *
BMI (kg/m^2^)	34.19±5.93	28.56±5.27	26.79±4.43	25.25±3.80	24.51±3.63	25.13±3.65	25.42±4.04	< 0.05
Diabetic parameters								
HbA1c (%)	8.79±1.76	7.57±1.21	6.61±1.16	6.34±1.07	6.24±1.22	6.24±1.16	6.46±1.08	< 0.05
HOMA-IR	11.23±14.94	6.14±16.00	2.64±3.10	2.37±3.86	1.58±15.00	2.96±7.51	2.17±1.93	< 0.05
Fasting blood sugar (mg/dl)	186.26±69.35	136.03±47.42	124.58±46.82	112.82±40.32	104.03±25.28	106.68±26.57	117.63±42.96	< 0.05
Insulin (μU/ml)	25.61±39.13	15.42±40.23	7.65±6.06	7.51±7.66	20.51±68.16	10.52±16.05	8.42±7.58	< 0.05
C-peptide (mg/ml)	3.14±1.86	1.99±0.79	2.00±0.93	1.46±0.57	1.58±0.98	1.43±0.55	1.93±1.46	< 0.05
Blood pressure								
SBP (mmHg)	136.22±15.44	125.95±16.85	124.32±17.69	122.65±16.15	117.09±14.24	123.59±18.12	123.85±22.32	< 0.05
DBP(mmHg)	88.78±12.05	81.14±12.69	77.00±11.91	74.91±10.88	70.65±10.93	74.19±14.26	72.79±14.26	< 0.05
Lipid profile								
T-CHO (mg/dl)	198.05±34.31	188.73±49.03	188.25±34.74	183.57±44.14	187.88±45.38	169.97±31.39	182.83±40.83	0.0697
HDL (mg/dl)	41.84±9.14	37.66±8.76	40.11±8.67	45.04±9.13	50.06±10.33	48.31±16.71	52.26±13.39	< 0.05
LDL (mg/dl)	114.80±30.35	121.85±36.99	120.22±35.00	114.91±35.82	115.01±38.46	104.15±26.68	111.55±33.70	0.6383
TG (mg/dl)	270.67±200.53	145.43±72.67	131.47±62.44	122.58±78.99	114.55±87.68	97.94±54.23	112.68±104.60	< 0.05
Liver function								
ALT (IU/l)	46.85±42.83	53.71±34.75	36.26±22.62	32.05±23.49	35.62±34.74	29.00±18.57	26.11±14.69	< 0.05
GGT (IU/l)	54.45±46.97	39.81±33.34	36.00±57.38	25.24±18.47	32.60±47.78	19.82±12.09	29.40±51.00	< 0.05
Albumin (g/dl)	4.39±0.30	4.20±0.28	4.26±0.24	4.19±0.40	4.17±0.37	4.24±0.23	4.24±0.37	0.0658
Hb (g/dl)	14.34±1.60	13.55±1.62	13.48±1.88	13.38±1.67	13.06±1.68	12.99±1.97	12.88±2.29	< 0.05
MCV (fl)	81.58±9.88	82.54±6.06	81.90±7.16	82.86±6.91	83.40±6.37	82.31±7.53	79.79±13.45	0.4734
Serum iron (μg/dl)	87.07±33.02	67.81±27.61	80.47±30.57	90.58±35.46	96.04±50.92	103.86±48.44	80.29±41.80	0.4326
TIBC (μg/dl)	332.73±66.22	301.77±64.57	308.61±47.78	316.73±67.22	326.81±76.27	329.26±49.79	333.98±81.48	0.9411
Ferritin (ng/ml)	193.81±161.67	273.52±301.51	178.73±144.03	160.12±130.51	129.56±111.08	122.05±144.19	74.31±67.05	< 0.05

* Denotes comparison between values ​​before surgery and 3 years after surgery. SADJB-SG: single-anastomosis duodenojejunal bypass with sleeve gastrectomy; SBP: systolic blood pressure; DBP: diastolic blood pressure; T-CHO: total cholesterol; HDL: high-density lipoprotein; LDL: low-density lipoprotein; TG: triglyceride; ALT: alanine aminotransferase; GGT: gamma glutamyl transferase; HbA1c: glycated hemoglobin; HOMA-IR: homeostatic model assessment for insulin resistance; MCV: mean corpuscular volume; TIBC: total iron binding capacity.

### OAGB

As shown in Table 3, the laboratory dataset outlines the preoperative and postoperative changes in various health parameters for individuals undergoing OAGB surgery over a 3-year period. OAGB results in a sustained reduction in BMI from 41.37±8.20 initially to 27.25±4.26 after 3 years (*P*<0.05). This substantial weight loss is accompanied by pronounced improvements in glycemic control, as reflected by a noteworthy drop in HbA1c levels from 7.30±1.85 to 5.50±0.82 during the same period (*P*<0.05). Furthermore, OAGB demonstrates enhanced insulin sensitivity, evidenced by a significant decrease in HOMA-IR values (*P*<0.05). The cardiovascular benefits of OAGB are evident through favorable reductions in both SBP and DBP over the 3-year period (*P*<0.05). Additionally, the surgery induces positive changes in the lipid profile, with decreases in T-CHO, LDL, and TG levels, coupled with an increase in HDL levels (*P*<0.05), indicative of improved lipid metabolism and reduced cardiovascular risk. OAGB’s impact also extends to liver function, as seen in significant improvements in ALT and GGT markers over the 3-year period (*P*<0.05). Meanwhile, microcytic anemia, resulted from iron deficiency, is noted. In summary of Table 3, OAGB not only leads to sustained weight loss but also demonstrates positive effects on glycemic control, insulin sensitivity, blood pressure, lipid profile, and liver function.

### SADJB-SG

Table 4 delves into the lasting effects of SADJB-SG on the exact health parameters as Table 3, spanning the preoperative phase and extending up to 3 years postoperatively. First, SADJB-SG results in a significant reduction in BMI, starting at 34.19±5.93 before the procedure and progressively decreasing to 25.42±4.04 after 3 years (*P*<0.05). Second, notable improvements in glycemic control are observed in diabetic parameters, with HbA1c levels decreasing from 8.79±1.76 preoperatively to 6.46±1.08 after 3 years, indicating improved glycemic control (*P*<0.05). Third, enhanced insulin sensitivity is noted as HOMA-IR values decreased significantly from 11.23±14.94 to 2.17±1.93 after 3 years, reflecting improved insulin sensitivity (*P*<0.05). Fourth, a reduction in blood pressure after SADJB-SG is documented over the 3-year period (*P*<0.05), indicating positive cardiovascular effects. Fifth, improvements in the lipid profile, such as a decreasing trend in T-CHO and an increased level of HDL (*P*<0.05), are both noted. Meanwhile, TG levels also decrease, indicating improved lipid metabolism. Sixth, liver function markers (ALT, GGT) demonstrate improvement, with significant reductions was observed (*P*<0.05). Seventh, similar to OAGB, iron deficiency anemia is also recognized in SADJB-SG group. In brief, Table 4 demonstrated positive effects of SADJB-SG on sustained weight loss, glycemic control, insulin sensitivity, blood pressure, lipid profile, and liver function.

**Figure 3:**
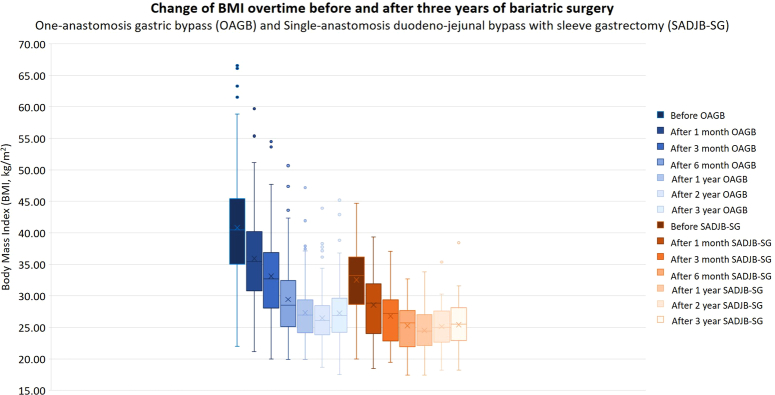
Change of BMI over time before and after three years of bariatric surgery: one-anastomosis gastric bypass (OAGB) and single-anastomosis duodenal-jejunal bypass with sleeve gastrectomy (SADJB-SG).

**Figure 4:**
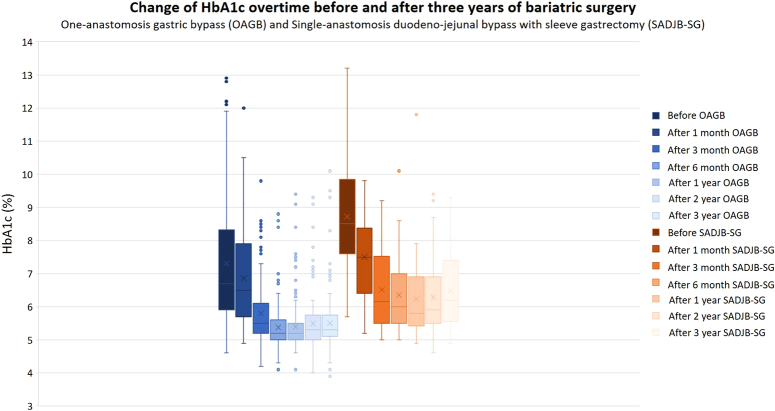
Change of HbA1c over time before and after three years of bariatric surgery: one-anastomosis gastric bypass (OAGB) and single-anastomosis duodenal-jejunal bypass with sleeve gastrectomy (SADJB-SG).

In conclusion, the 3-year evaluation of OAGB and SADJB-SG surgeries, as delineated in Tables 3 and 4, underscore sustained and substantial improvements in health. Both procedures demonstrate lasting advantages, encompassing weight loss, enhanced glycemic control, improved insulin sensitivity, positive cardiovascular impacts, and favorable changes in lipid and liver function. Notably, a minor rebound in BMI and HbA1c is observed in both cohorts during the first and second postoperative years, as shown in Figure 3 and Figure 4. These rebound-effects are thought to be associated with inadequate lifestyle modification of the individuals after the patients undergo primary MBS.

### Prediction of 10-year and lifetime risks of CHD, T2D, HTN, stroke, MACE before and after 3 years of surgery

The secondary objective of this research is to evaluate changes in the risks of CHD, T2D, HTN, stroke, MACE over a 10-year span, a crucial consideration in the prompt management of obesity and its related comorbidities. Table 5 presents the 10-year risks of the above concerns before and after 3 years for individuals undergoing either OAGB or SADJB-SG, calculated with using the Taiwan MACE risk prediction model. For CHD, both surgeries significantly reduced the overall risk after 3 years, with a decreased tendency from 23.0 to 9.0% (OAGB, *P*<0.001) and 15.90 to 8.40% (SADJB-SG, *P*<0.01), respectively. In terms of T2D, both surgeries demonstrate significant reductions in overall risk, with OAGB leading to a substantial decrease from 89.0 to 29.3% (*P*<0.001). Notably, SADJB-SG shows a significant shift from 94.3 to 62.0% (*P*<0.001). HTN risks are also reduced significantly after 3 years for both surgeries, with OAGB resulting in a decrease from 85.0 to 54.4% (*P*<0.001) and SADJB-SG bringing out a drop from 72.90 to 49.80% (*P*<0.001). The risk of stroke significantly decreases after 3 years for both procedures. OAGB presents an improvement from 54.0 to 8.5% (*P*<0.001), while SADJB-SG exhibits a reduction from 21.30 to 10.60% (*P*<0.001). Similarly, MACE risks significantly decrease after 3 years for both surgeries. OAGB leads to an amelioration from 27.0 to 12.4% (*P*<0.001), whilst SADJB-SG results in a reduction from 21.20 to 12.00% (*P*<0.001).

**Table 5:** Prediction of 10-year and lifetime risks of CHD, T2D, HTN, stroke, MACE risk before and after 3 years of surgery: OAGB and SADJB-SG.

10-year risks of CHD, HTN, DM, stroke, and MACE, calculated with Taiwan MACE risk prediction model						
	OAGB Before			SADJB-SG		
	3-year	*P*	Before	3-year	*P*	
CHD						
Overall risk (%)	23.0%	9.0%	< 0.001	15.9%	8.4%	< 0.01
Low risk (<10%), *n* (%)	63 (35.6%)	141 (79.7%)		20 (42.6%)	30 (63.8%)	
Moderate risk (10–20%), *n* (%)	40 (22.6%)	26 (14.7%)		14 (29.8%)	14 (29.8%)	
High-risk (20–30%), *n* (%)	29 (16.4%)	5 (2.8%)		7 (14.9%)	3 (6.4%)	
Very high-risk (>30%), *n* (%)	45 (25.4%)	5 (2.8%)		6 (12.8%)	0 (0.0%)	
T2D						
Overall risk (%)	89.0%	29.3%	< 0.001	94.3%	62.0%	< 0.001
Low risk (<10%), *n* (%)	1 (0.6%)	18 (10.2%)		1 (2.1%)	5 (10.6%)	
Moderate risk (10–20%), n (%)	1 (0.6%)	31 (17.5%)		0 (0.0%)	7 (14.9%)	
High-risk (20–30%), *n* (%)	3 (1.7%)	89 (50.8%)		0 (0.0%)	4 (8.5%)	
Very high-risk (>30%), *n* (%)	171 (96.6%)	39 (22.0%)		46 (97.9%)	31 (66.0%)	
HTN						
Overall risk (%)	85.0%	54.4%	< 0.001	72.9%	49.8%	< 0.001
Low risk (<10%), *n* (%)	7 (4.0%)	7 (4.0%)		3 (6.4%)	3 (6.4%)	
Moderate risk (10–20%), *n* (%)	1 (0.6%)	16 (9.0%)		2 (4.3%)	9 (19.1%)	
High-risk (20–30%), *n* (%)	6 (3.4%)	13 (7.3%)		2 (4.3%)	1 (2.1%)	
Very high-risk (>30%), *n* (%)	163 (92.1%)	141 (79.7%)		40 (85.1%)	34 (72.3%)	
Stroke						
Overall risk (%)	54.0%	8.5%	< 0.001	21.3%	10.6%	< 0.001
Low risk (<10%), *n* (%)	58 (32.8%)	141 (79.7%)		15 (31.9%)	25 (53.2%)	
Moderate risk (10–20%), *n* (%)	43 (24.3%)	24 (13.6%)		14 (29.8%)	18 (38.3%)	
High-risk (20–30%), *n* (%)	24 (13.6%)	8 (4.5%)		5 (10.6%)	2 (4.3%)	
Very high-risk (>30%), *n* (%)	52 (29.4%)	4 (2.3%)		13 (27.7%)	2 (4.3%)	
MACE						
Overall risk (%)	27.0%	12.4%	< 0.001	21.2%	12.0%	< 0.001
Low risk (<10%), *n* (%)	44 (24.9%)	59 (33.3%)		12 (25.5%)	20 (42.6%)	
Moderate risk (10–20%), *n* (%)	40 (22.6%)	95 (53.7%)		14 (29.8%)	19 (40.4%)	
High-risk (20–30%), *n* (%)	30 (16.9%)	13 (7.3%)		12 (25.5%)	7 (14.9%)	
Very high-risk (>30%), *n* (%)	63 (35.6%)	10 (5.6%)		9 (19.1%)	1 (2.1%)	
10-year and lifetime risks of atherosclerotic cardiovascular disease (ASCVD), calculated with the China-PAR project model						
	OAGB			SADJB-SG		
	Before	After 3-year	*P*	Before	After 3-year	*P*
Lifetime risk of atherosclerotic cardiovascular disease						
Overall risk (%)	33.7%	14.3%	< 0.001	32.1%	15.2%	< 0.001
Low risk (<32.8%), *n* (%)	98 (55%)	174 (98%)		28 (60%)	46 (98%)	
High-risk (≧32.8%), *n* (%)	79 (45%)	3 (2%)		19 (40%)	1 (2%)	
10-year risk of atherosclerotic cardiovascular disease						
Overall risk (%)	5.7%	2.2%	< 0.001	4.8%	2.6%	< 0.01
Low risk (< 5%), *n* (%)	97 (55%)	157 (89%)		32 (68%)	40 (85%)	
Moderate risk (5–9.9%), *n* (%)	47 (27%)	19 (11%)		11 (23%)	6 (13%)	
High-risk (> 10%), *n* (%)	33 (19%)	1 (1%)		4 (9%)	1 (2%)	

CHD, coronary heart disease; HTN, hypertension; MACE, major adverse cardiovascular events; OAGB, one anastomosis gastric bypass; SADJB-SG, single-anastomosis duodenojejunal bypass with sleeve gastrectomy; T2D, type 2 diabetes mellitus.

Moreover, Table 5 also includes 10-year and lifetime risks of atherosclerotic cardiovascular disease (ASCVD) using the China-PAR project model. Both surgeries significantly reduce the lifetime and 10-year risks, with OAGB and SADJB-SG showing substantial risk reduction. In summary, the findings underscore the substantial and statistically significant improvements in cardiovascular risk factors following both OAGB and SADJB-SG surgeries, supporting their efficacy in reducing the long-term risks of CHD, HTN, T2D, stroke, and MACE.

## Discussion

Our study concludes that OAGB and SADJB-SG have similar long-term therapeutic effects on obesity, T2D, HTN and dyslipidemia aligned with existed literature^21,22^. On top of that, MBS has been demonstrated as having a notable protective influence on CVD^4,10,13,23,24^. Individuals with MBS history has reduced mortality risk following a MI compared to non-MBS controls^25^. Additionally, MBS is linked to a diminished risk of MACE in individuals grappling with obesity and T2D^23^. In a retrospective observational matched cohort study, based on the US population, involving 20 235 patients (surgical and nonsurgical), aged 50 on average, with a higher proportion of females, with baseline mean BMI around 44 kg/m^2^, bariatric surgery demonstrated a lower composite incidence of macrovascular events and coronary artery disease at 5 years compared to nonsurgical interventions^26^. A Swedish observational matched cohort study revealed that individuals with severe obesity and a history of MI, who underwent MBS, showed a decreased risk of mortality and recurrent MI compared to matched controls who did not undergo MBS^27^. A population-based cohort study conducted in Ontario, Canada, evaluated the long-term impact of bariatric surgery on MACE in patients with obesity, T2D, and/or HTN. This study, based on Canadian population, found that among patients with obesity, T2D, and HTN, MBS is associated with a sustained (≥10 years) decrease in the incidence of MACE. The 10-year absolute risk reduction for the surgical group was 5.14%^23^. A retrospective, matched, controlled cohort study of The Health Improvement Network primary care database, based on the UK population, examined 5170 exposed (treated with MBS) and 9995 control (nonsurgical intervention) participants, MBS group showed a 20.0% mean weight loss, with gastric bypass linked to a significantly lower cardiovascular disease risk. MBS was also associated with reduced all-cause mortality, HTN, and heart failure^28^.

Despite the aforementioned studies examining the impact of MBS on cardiovascular outcomes, there is a lack of solid evidence investigating the sustainable long-term impact of MBS on major cardiovascular events in the Asian population. The Taiwanese population comprises Han Chinese (95%), indigenous Malayo-Polynesian peoples (2.5%), and new immigrants from China and Southeast Asia (2.5%). Moreover, duodenal exclusion procedures such as OAGB and SADJB-SG have been proven to be effective novel MBS for treating patients with obesity and T2D^29,30^. However, there are limited studies investigating impacts of OAGB and SADJB-SG with a focus on long-term prediction risks of cardiovascular events. Therefore, our prospectively-collected retrospective observational study provides key evidence regarding the association between MBS and the alteration of 10-year and lifetime risk of MACE risks before and after 3 years of surgery (OAGB and SADJB-SG) in the Asian population by using two different risk assessment models, derived from the national database of both Taiwan and China.

This study has some limitations. Firstly, this retrospectively analyzed observational study may not share the similar level of evidence as randomized controlled trials, even though the data were prospectively-collected. However, despite this methodological concern, retrospective studies are still considered essential in reflecting actual clinical aspects. Secondly, this study reflects a common phenomenon in MBS research: a relatively low follow-up rate^31,32^. Nonetheless, a relatively low follow-up rate, resulted from data missing completely at random, may still provide valuable information to clinical practice and bring potential insights to future study design. Our study is conducted with cohorts having complete data for a period of three years, the concern about the probability of under-representation resulting from missing data shall be carefully examined. Thus, to validate the reliability of our results, we include all patients, incorporating the censor time, to inspect the change in BMI and HbA1c of both OAGB and SADJB-SG cohorts (Supplementary Figures 1 to 4, Supplemental Digital Content 2, http://links.lww.com/JS9/C631, Supplemental Digital Content 3, http://links.lww.com/JS9/C632, Supplemental Digital Content 4, http://links.lww.com/JS9/C633, Supplemental Digital Content 5, http://links.lww.com/JS9/C634). Notably, despite the concern about the potential effect of missing data, a similar trend before and after 3 years of MBS (OAGB and SADJB-SG) is observed among the results of both arms: ‘all patients with censor time’ and ‘the cohorts with complete data of a 3-year follow-up’. Hence, this auxiliary finding implies that our data-completeness approach does not greatly limit the validity of our current results. In spite of the above limitations, our study possesses several strengths. Firstly, we deliberately choose to investigate the less-commonly-examined MBS procedures, namely OAGB and SADJB-SG, conducting a retrospective analysis of patient cohorts. This analysis is based on the premise of the completeness of follow-up objective laboratory data. Secondly, the duration of follow-up time, spanning 3 years after surgery, exceeds the temporal scope of a majority of existing clinical retrospective studies on MBS within the Asian population. Thirdly, we employ risk assessment tools tailored to the ethnic characteristics of the Taiwanese population to analyze the risk of MACE. Lastly, our study contributes valuable insights into how the Asian population, particularly those with obesity and metabolic comorbidities, can derive benefits from MBS.

## Conclusions

Our results reinforce existing associations and contribute new insights to the current body of knowledge, with a specific focus on relatively rarely-covered procedures, including OAGB and SADJB-SG. Among the patients selected for the current study, based on the spirit of personalized surgical plan, each patient receive informative discussions before their primary surgery regarding the choice of MBS, OAGB, or SADJB-SG. For those with less severe obesity but complicated by more worrisome severity of T2D, SADJB-SG is considered a reasonable choice due to the more powerful control of diabetes through the duodenal switch. To conclude, OAGB and SADJB-SG demonstrate sustained 3-year improvements in weight reduction, remission of T2D, and obesity-related comorbidities, as well as significant reductions in 10-year MACE, stroke, and ASCVD risks.

## Disclosure

In adherence to ethical standards, we affirm the absence of conflicts of interest, financial or otherwise. All contributors meet authorship criteria, and research funding sources, if any, are transparently acknowledged to ensure scientific integrity and transparency.

## Ethical approval

Ethical approval was given by Institutional Review Board of Min-Sheng General Hospital (MSIRB2014009), Tri-Service General Hospital (B202105089).

## Consent

Written informed consent was obtained from the patient for publication. A copy of the written consent is available for review by the Editor-in-Chief of this journal on request.

## Source of funding

None.

## Author contribution

K.-F.H.: conceptualization, methodology, writing – review and editing, and supervision; H.-M.P.: data curation, software, visualization, and writing – original draft; W.-J.L.: data resources and curation, and investigation; K.-H.S.: data curation and software; T.-C.S.: analysis; M.-H.L.: methodology; C.-H.L.: data curation; K.-F.H. and H.-M.P.: completed the manuscript with input from all authors.

## Conflicts of interest disclosure

The authors declare no conflicts of interest.

## Research registration unique identifying number (UIN)

ClinicalTrials.gov ID: NCT06254339.

## Guarantor

Kuo-Feng Hsu (hsukf97@ndmctsgh.edu.tw), Division of General Surgery, Department of Surgery/ Bariatric and Metabolic Surgery and Weight Management Center, Tri-Service General Hospital.

## Data availability statement

Data will be available from the corresponding author when required.

## Provenance and peer review

Not commissioned, externally peer-reviewed.

## Supplementary Material

**Figure:**
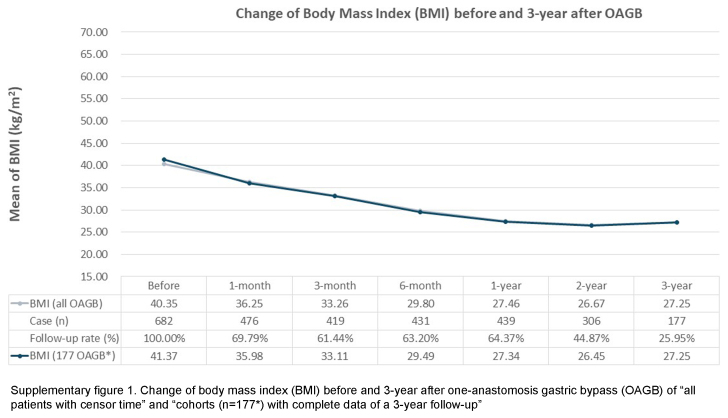


**Figure:**
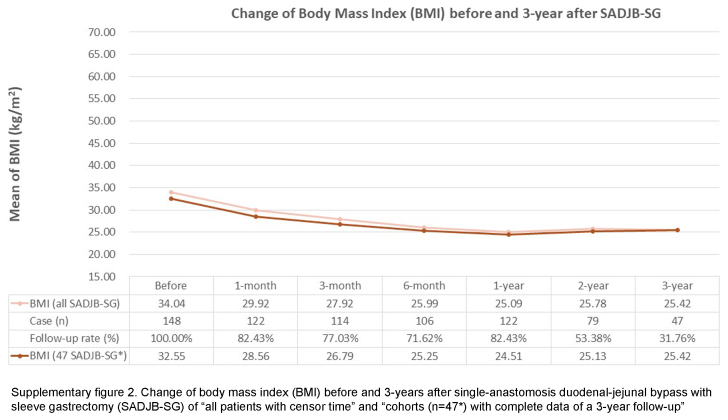


**Figure:**
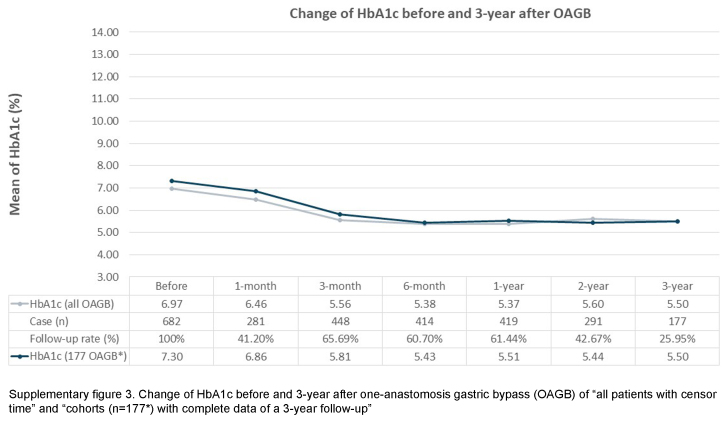


**Figure:**